# Preventive role of probiotic bacteria against gastrointestinal diseases in mice caused by *Giardia lamblia*

**DOI:** 10.1042/BSR20204114

**Published:** 2021-02-22

**Authors:** Wafa A. Al-Megrin, Shadia H. Mohamed, Moudy M. Saleh, Hany M. Yehia

**Affiliations:** 1 Department of Biology, Faculty of Science, Princess Nourah bint Abdulrahman University, Saudi Arabia; 2 Department of Zoology, Faculty of Science, Ain Shams University, Cairo, Egypt; 3 Department of Zoology, Faculty of Science, King Saud University, Riyadh, Saudi Arabia; 4 Department of Food Science and Nutrition, College of Food and Agriculture Science, King Saud University, Riyadh 11451, Saudi Arabia; 5 Department of Food Science and Nutrition, Faculty of Home Economics, Helwan University, Cairo 11221, Egypt

**Keywords:** Giardia lamblia, ELISA, histopathological studies, probiotics

## Abstract

Giardiasis is one of the most prevalent gastrointestinal diseases in the world. It is caused by *Giardia, Giardia lamblia*, a common and opportunistic zoonotic parasite. The aim of our work is to find a natural and safe alternative treatment for giardiasis, specifically, to determine if probiotic bacteria (*Lactobacillus acidophilus, Bifidobacterium bifidum*, and *Lactobacillus helveticus*) can contribute to treatment, and act as preventives. Sixty weanling albino mice, *Mus musculus*, were divided into control and experimental, probiotic-fed groups. We determined infection intensity, and cure and prevention rates of giardiasis through ELISA (enzyme-linked immunosorbent assay) of stool samples and histopathological comparison of intestinal tissue. In experimental groups, there was a significant reduction in infection intensity (*P*<0.001) on days 10, 15, and 20, while cure rate reached 87.5%. The control group showed no signs of reduced infection or cure and only the group treated with probiotics prior to infection showed significant prevention rates. In the experimental groups, intestinal changes due to giardiasis appeared 7 days post-infection. However, almost all of these changes disappeared by the 25th day. Our results suggest a beneficial and significant effect of probiotics in the prevention and treatment of giardiasis in mice.

## Introduction

Giardia, *Giardia lamblia*, causing giardiasis, is the most frequent parasite reported in children and the most frequently found protozoan in water samples [1,2]. It primarily affects children and the immunosuppressed and exists in two interconvertible forms: cyst and trophozoite. Infection is acquired by ingestion of food or water contaminated with cysts, which become trophozoites that colonize the small intestine by attachment to the epithelial microvillus [3,4]. Some infected people present gastrointestinal symptomatology; however, in many cases the disease is completely asymptomatic. In such cases, patients act as carriers, releasing large numbers of cysts into the environment and promoting the transmission of infection [5].

There are different techniques to detect *G. lamblia*, the most common being microscopic examination of stool specimens. However, this technique can give a false negative result. Detection of the parasite antigen in stool specimens through ELISA (enzyme-linked immunosorbent assay) using monoclonal antibodies is a more sensitive and accurate method [6].

There are several pharmaceutical agents used in the treatment of giardiasis including metronidazole [7], albendazole, tinidazole, and nitazoxanide [8]. Some of these are administered in one dose while others (such as metronidazole) require several doses each day for 2–3 days. In refractory cases, through resistance or reinfection, the combination of two or more drugs can be necessary [9,10]. All such drugs can produce collateral effects like nausea, metallic taste, yellow skin pigmentation, hepatic damage, inefficient treatment, and resistance [11]. Two major reasons to search for new giardiasis treatments are the increase in the number of resistant strains and the need to provide alternatives that require only one dose and have high efficiency with fewer side effects. Natural medicine is a viable alternative [12]. Some natural products have been extracted from plants in India and Africa and used against *G. lamblia* with up to 98% recovery rates [13]. A combination of 5-nitroimidazole and albendazole or mebendazole, and quinacrine monotherapy, are rational choices in nitroimidazole refractory infections, but randomized controlled studies are needed. Further research into more recent clinical isolates is necessary to uncover mechanisms for the increase in metronidazole refractory giardiasis observed during the last decade [14].

Biotherapy is a type of natural alternative medicine that uses probiotics, including useful bacteria that inhabit the small intestine of humans. Antibiotics cause an imbalance in bacterial communities of the small intestine, resulting in parasites being better able to attack the mucosa [15]. Studies have shown that beneficial bacteria such as *Lactobacillus* spp. and *Bifidobacterium* spp. are the most important types commonly used in practice [16]. Studies have confirmed that these bacteria maintain the natural balance of the bacterial system in human intestines via various mechanisms. These include acting as a barrier to prevent the passage of pathogens through the mucus layer, producing antimicrobial agents, and competing with pathogens for food and adhesion sites [17]. These types of bacteria have shown their importance in increasing immunity as well as resistance to diseases. Some mechanisms through which this occurs are now well identified. However, some others require further research and study [18]. The interaction between the intestinal microbiota and immune system will be of great importance to the field of *Giardia* pathogenesis. Several studies have identified mechanisms whereby intestinal bacteria can influence the development of Th17 and Treg immune responses [19–22]. Intestinal microbiota clearly impact colonization and pathogenesis of *Giardia* and recent work highlights potential influences on the anti-*Giardia* immune response as well. At steady state, there is complex cross-talk between microbes and host cells and it is becoming increasingly evident that *Giardia* infections have the capability to perturb microbial homeostasis, causing microbial dysbiosis. Since *Giardia* infections can lead to post-infectious IBS and CFS, it will be interesting to understand the long-lasting effects of *Giardia*-induced microbial dysbiosis in promoting these post-infectious disease syndromes. Finally, since many advances in our understanding of protective *Giardia* immunity have been discovered using murine infection models or human cell culture models, additional emphasis on studies of immune responses in human infections are needed. The greater challenge for our field resides in translating these advances towards humans systems to generate novel therapeutics that promote human health.

The aim of the present study was to find a natural and safe alternative treatment for giardiasis. The impact of bacteria (*Lactobacillus acidophilus, Bifidobacterium bifidum*, and *Lactobacillus helveticus*), in the treatment of giardiasis was considered, and the potential extent of the preventive role of probiotics examined.

## Materials and methods

### Experimental animals and ethics protocol

The study was conducted on 60 albino mice (age: 3–4 weeks; weight: 20–25 g) and free from parasitic infections. They were obtained from VACSERA (Giza, Egypt). Prior to the experiment, the mice were housed in wire polypropylene cages under controlled environmental conditions (25 ± 2°C temperature, 55–60% humidity, and a 12-h light/dark cycle) for 2 weeks. All experiments were performed in accordance with the European Community Directive (95/701/EEC). The animal care procedures agreed with the National Institutes of Health (NIH) Guidelines for the Care and Use of Laboratory Animals, eighth edition, and were approved by the Institutional Animal Ethics Committee for Laboratory Animal Care at the Zoology Department, Faculty of Science, Helwan University (Approval number: 99 HU2020/Z/HY0420-01). To ensure that mice were free from intestinal parasites, stool samples were examined with direct smears using Lugol’s iodine for three consecutive days before starting the experiment [23]. All animals were killed by CO_2_ asphyxiation, dissected and small intestine collected as samples for biochemical and histopathological analyses were frozen at −80°C until processed.

Animals were divided into an experimental and control group.

The experimental group (I) was further divided into two, while the control group (II) was further divided into three. Each final grouping contained 12 mice.

#### Group IA

Starting 7 days before infection and throughout the research period, this group was fed daily with a mixture of beneficial bacteria strains consisting of *L. acidophilus, B. bifidum*, and *L. helveticus* (each contained 1 billion CFU/1 capsule), with citrus pectin powder (100 mg/capsule) (Mega Acidophilus supplement; GNC, Saudi Arabia). Daily doses were calculated against the dose GNC recommends for humans, according to body weight to each mouse.

#### Group IB

Starting from the seventh day of infection and throughout the research period, this group was fed daily as outlined for the previous group.

#### Group IIA

Infected with parasite and not fed with probiotics.

#### Group IIB

Fed with citrus pectin daily, starting 7 days before infection and throughout the research period.

#### Group IIC

Fed with citrus pectin daily, starting from the seventh day of infection and throughout the research period.

### Isolation of *G. lamblia* cysts from stool samples and preparation of the infective inoculum

*G. lamblia* cysts were obtained from fresh fecal samples of clinical patients at Helwan University Hospital in Helwan, Egypt and from fresh fecal samples of infected mice at the animal house at the College of Science, Helwan University. A microscopic examination of the wet surfaces of samples showed that there were at least 3–5 cysts per microscopic field (×400). The study was carried out in accordance with The Code of Ethics of the World Medical Association (Declaration of Helsinki) and that all subjects provided informed consent.

Fresh, positive samples were treated by emulsification in a physiological saline solution (0.85%) and sieved using stainless steel filters (20 holes/cm^2^) to remove crumbs and allow the *G. lamblia* cysts to pass through. Cysts were then concentrated using a centrifuge (2000 rpm, for 3 min); this was followed by repeated washing using physiological saline solution. Finally, the precipitate was collected with drops of physiological solution and the cysts were suspended in the solution at a concentration of 500000 cysts/ml (controlled using a counting slide).

### Method of infection

Infection was induced in the mice by feeding the parasite suspension (0.2 ml/mouse) with a (10^5^) dose bag/mouse (Vinayak et al., 2015 [24]), using a 1-ml syringe after removal of the needle. To ensure infection, stools were collected and dyed with Lugol’s iodine from the day after feeding the parasite suspension, until the fifth day.

### Parasitological studies

#### Determination of infection intensity

Infection intensity was calculated on days 10, 15, and 20 following infection in the following manner:

1. The fecal pellets of each mouse were collected within a 2-h period in a glass tube. Each tube was labeled to show the sample data and contained drops of physiological saline solution. Tubes were kept in the refrigerator [25].
2. Cysts were isolated using the formalin and ether deposition method. The precipitate was collected in 1 ml of physiological saline solution, and wet, dyed swabs were processed on a count plate. The number of cysts was calculated in ten microscopic fields (400 × 400) for each mouse fecal sample.

#### Determination of protection and cure rates

The presence or absence of *G. lamblia* antigen was determined in feces on days 5, 7, 14, 21, and 28 after infection by ELISA using monoclonal antibodies (ProSpecT Giardia Microplate Assay; Remel, **Oxoid Limited** Wade Road Basingstoke Hampshire RG24 8PW, United Kingdom).

#### Calculation of recovery and prevention rate

The cure rate was determined using the following equation:

$$\text{Cure rate}=\frac{\text{Number of cured mice}}{\text{Number of treated mice}}\times 100\;$$

The recovery of mice was measured according to the results of ELISA. The rate of prevention was calculated using the following equation:

$$\text{Prevention rate}=\frac{\text{Number of uninfected mice}}{\text{Number of infected mice}}\times 100$$

Infection was indicated by the presence of cysts in the stool, as identified through wet swabs and ELISA results. The number of cysts was calculated by taking the average of cysts per mouse in each field of microscopy plus the standard deviation.

#### Histopathological studies

Histopathological changes were studied in the small intestine by taking 3–4 cm of the upper part of the jejunum from groups IA, IB, and IIA. Samples from three mice in each group were studied after 10 and 25 days of infection and compared with segments of healthy mice of the same age and size. Samples were collected and stabilized in (10%) formalin, processed in wax sections, and dyed with Hematoxylin and Iodine.

### Statistical analysis

Statistical analysis of the results was carried out using SPSS and included the following statistical methods: arithmetic mean, standard deviation and one-way ANOVA, known as F-distribution. Values calculated using these statistical methods could be classified according to their degree of probability, *P*, as follows: *P*≤0.05 is significant, *P*≤0.01 is of high significance, *P*≤0.001 is of very high significance, and *P*≥0.05 is considered insignificant.

## Results

### Parasitological results

#### Intensity of infection

**Figure 1:**
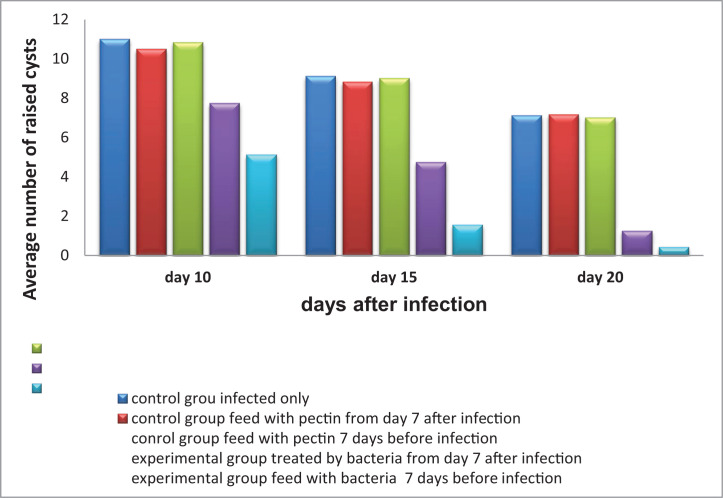
The incidence of *Giardia* cysts In the stool from different study groups during infection.

The results of the control group infected with *G. lamblia* only (Group IIA) showed a statistically significant decrease (*P*<0.05) in the number of cysts in the feces on day 20 (1.45 + 7.12) compared with day 10 (1.51 + 11), while no significant differences were observed between days 15 (1.12 + 9.12) and 10 or 20. In control group IIC, no significant differences were observed among days 10 (1.04 + 10.50), 15 (1.47 + 8.83), and 20 (1.47 + 7.17). Control group IIB showed a significant decrease (*P*<0.05) in the number of cysts in the stool on day 20 (1.41 + 7.00) compared with day 10 (1.16 + 10.83). However, no significant differences were found between day 15 (0.89 + 9.00) compared with days 10 or 20 (Table 1 and Figure 1).

**Table 1:** Number of *G. lamblia* cysts in feces from different groups during the infection period

Groups	Number of cysts			Statistical analysis
	Day 20	Day 15	Day 10	
Group IIA	1.51 ± 11.00	1.12 ± 9.12	1.45 ± 7.12	gGF = 15.8 *P*<0.001
Group IIC	1.04 ± 10.50	1.47 ± 8.83	1.47 ± 7.17	F = 9.2 *P*<0.001
Group IIB	1.16 ± 10.83	0.89 ± 9.00	1.41 ± 7.00	F = 15.8 *P*<0.001
Group IB	1.28 ± 7.75	0.66 ± 4.75	0.70 ± 1.25	F = 82.6 *P*<0.0001
Group IA	0.21 ± 5.14	0.78 ± 1.57	0.53 ± 0.43	F = 80.6 *P*<0.0001

The results of the experimental group IB showed a decrease in the number of cysts in the stool on day 20 (0.70 + 1.25) compared with days 10 (1.28 + 7.75) and 15 (0.66 + 6.75) with very high significance (*P*<0.0001). Additionally, a significant decrease was detected on day 15 compared with day 10 (*P*<0.05). The results also showed a highly significant decrease (*P*<0.001) in the number of cysts in the experimental group IA on day 10 (0.21 + 6.14) compared with days 15 (0.78 + 1.57) and 20 (0.53) +0.43), along with a significant decrease on day 15 compared with day 20.

In comparing control groups, the results showed no significant decrease (*P*>0.05) between them on days 10, 15 and 20, and a continued decrease in the number of cysts. Contrastingly, the comparison of experimental groups showed significant differences (*P*<0.05) on each of the days 10, 15, and 20 when compared with each other.

#### Protection and cure rates

Statistical analyses for the results of *G. lamblia* antigen detection in the stool by using ELISA indicated very high significant differences (*P*<0.001) in all three control groups on days 5 and 7 compared with the remaining days of the examination. On day 14, the differences were insignificant (*P*>0.05), while days 17 and 21 showed significant differences (*P*<0.05) compared with day 28. Furthermore, there were insignificant differences among days 17, 21, and 28 (Table 2).

**Table 2:** Statistical analyses of the detection of *G. lamblia* antigen in feces on different days for each group

Control groups									Experimental groups					
IIA			IIC			IIB			IB			IA		
Day A	Day B	Statistical analysis	Day A	Day B	Statistical analysis	Day A	Day B	Statistical analysis	Day A	Day B	Statistical analysis	Day A	Day B	Statistical analysis
5	7	*P*<0.0001	5	7	*P*<0.0001	5	7	*P*<0.0001	5	7	*P*<0.0001	5	7	*P*<0.001
	14	*P*<0.0001		14	*P*<0.0001		14	*P*<0.0001		14	*P*<0.05		14	*P*<0.001
	17	*P*<0.0001		17	*P*<0.0001		17	*P*<0.0001		17	*P*>0.05		17	*P*>0.05
	21	*P*<0.0001		21	*P*<0.0001		21	*P*<0.0001		21	*P*>0.05		21	*P*>0.05
	28	*P*<0.0001		28	*P*<0.0001		28	*P*<0.0001		28	*P*>0.05		28	*P*>0.05
7	14	*P*<0.0001	7	14	*P*<0.0001	7	14	*P*<0.0001	7	14	*P*<0.0001	7	14	*P*>0.05
	17	*P*<0.0001		17	*P*<0.0001		17	*P*<0.0001		17	*P*<0.0001		17	*P*<0.0001
	21	*P*<0.0001		21	*P*<0.0001		21	*P*<0.0001		21	*P*<0.0001		21	*P*<0.0001
	28	*P*<0.0001		28	*P*<0.0001		28	*P*<0.0001		28	*P*<0.0001		28	*P*<0.0001
14	17	*P*>0.05	14	17	*P*>0.05	14	17	*P*>0.05	14	17	*P*<0.05	14	17	*P*<0.001
	21	*P*>0.05		21	*P*>0.05		21	*P*>0.05		21	*P*<0.05		21	*P*<0.001
	28	*P*<0.05		28	*P*<0.05		28	*P*<0.05		28	*P*<0.001		28	*P*<0.001
17	21	*P*>0.05	17	21	*P*>0.05	17	21	*P*>0.05	17	21	*P*<0.05	17	21	*P*<0.05
	28	*P*>0.05		28	*P*>0.05		28	*P*>0.05		28	*P*>0.05		28	*P*>0.05
21	28	*P*>0.05	21	28	*P*>0.05	21	28	*P*>0.05	21	28	*P*>0.05	21	28	*P*>0.05

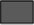
 significant 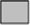 high significant 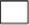 very high significant 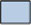 insignificant.

In experimental group IB, statistical analyses showed very high significant differences between day 7 (2.38 + 7.68) and the rest of the days of the examination. Additionally, on day 14 (0.81 + 2.12), there were significant differences with each of the days 5 (0.15 + 0.86), 17 (0.19 + 0.87), and 21 (0.20 + 0.76), along with a highly significant difference (*P*<0.01) with day 28 (0.24 + 0.58). On day 17, there were significant differences with day 21, while the differences were insignificant regarding the rest of the days.

In experimental group IA, the studies showed very high significant differences between day 7 (2.39 + 2.97) and the rest of the days of the examination, with the exception of day 14 (1.91 + 2.51) where the difference was insignificant. The differences were very highly significant between day 14 and the rest of the days of the examination, and on the day 17 (0.29 + 0.52) there was a significant with day 21 (0.33 + 0.41). All other comparisons between days were insignificant

The results of the detection of *G. lamblia* antigen in the stool by using monoclonal antibodies (ELISA) showed the beginning of a cure for the mice in both experimental groups on the 14th day after infection. The percentage of mice cured reached 87.5% on day 28. No cure was observed in the control groups.

Furthermore, in determining the preventive role of probiotics, the results showed that in experimental group IA, the rate of prevention was 43% on the seventh day. This rate dropped to 30% on the 14th day until the end of the experiment. There was no prevention identified in the rest of the groups, where the percentage of *G. lamblia* infection was 100%.

### Histopathological results

#### Histopathological changes in the jejunum of control group IIA

**Figure 2:**
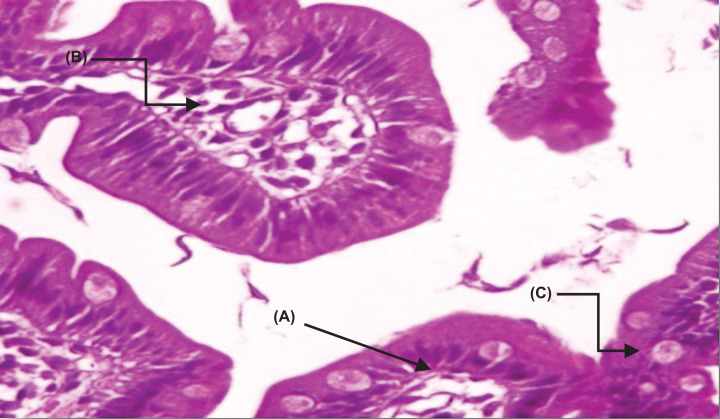
A section of jejunum from a mouse In control group IIA, (**A**) integration in some villi, (**B**) cellular infiltration, and (**C**) showing the density of the trophozoites between the cells and their adhesion to the mucous surface of cells (H & E, 400×).

The segmental examination showed a high frequency of trophozoites in the intestinal cavity and between the villi, attached to the surface of apparently damaged epithelial cells. There was also a slight increase in cellular infiltration, as well as clear changes in the shape of the villi, which appeared short and wide (Figure 2).

#### Histopathological changes in the jejunum of experimental group IB

**Figure 3:**
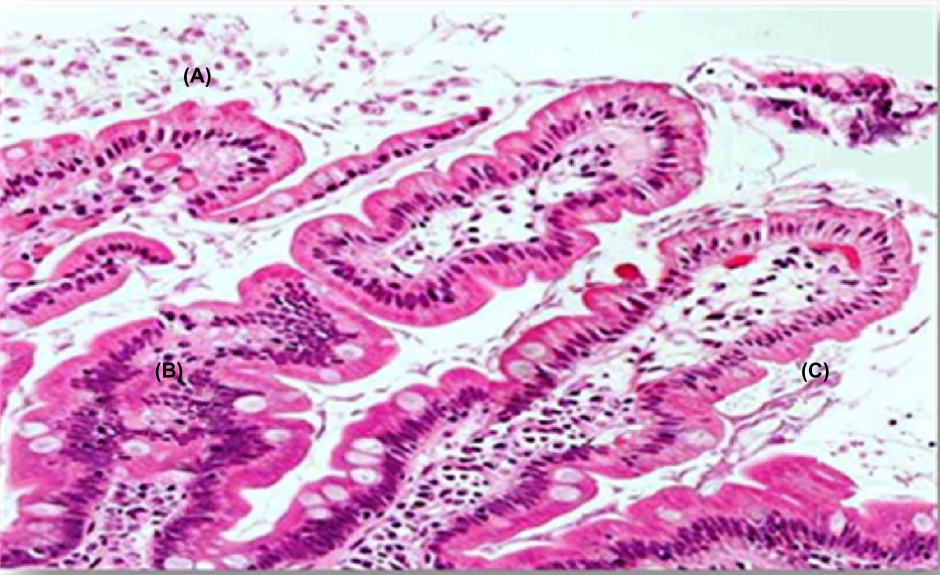
A section of jejunum from a mouse In experimental group IB, showing (**A**) integration in some villi, (**B**) increase in cellular infiltration, and (**C**) presence of some trophozoites (H & E, 400×).

**Figure 4:**
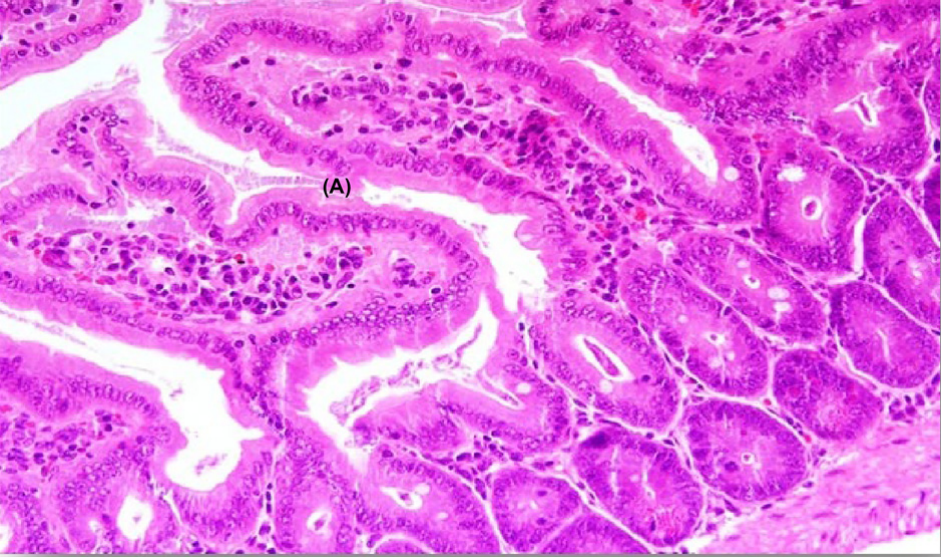
A section of jejunum from a mouse In experimental group IB on the 25th day, showing the (**A**) complete disappearance of trophozoites and that the villi is restored to its normal shape (H & E, 20×).

Segmental examination showed similar pathological changes to the untreated group but less severe. On the tenth day (Figure 3), there was a little or no presence of trophozoites. Additionally, there was no change in the shape of villi. There was a marked increase in cellular infiltration in the central pulp. On the 25th day (Figure 4), the presence of trophozoites disappeared completely and most of the mumps recovered their normal form, except for a slight increase in cellular infiltration.

#### Histopathological changes in the jejunum of experimental group IA

**Figure 5:**
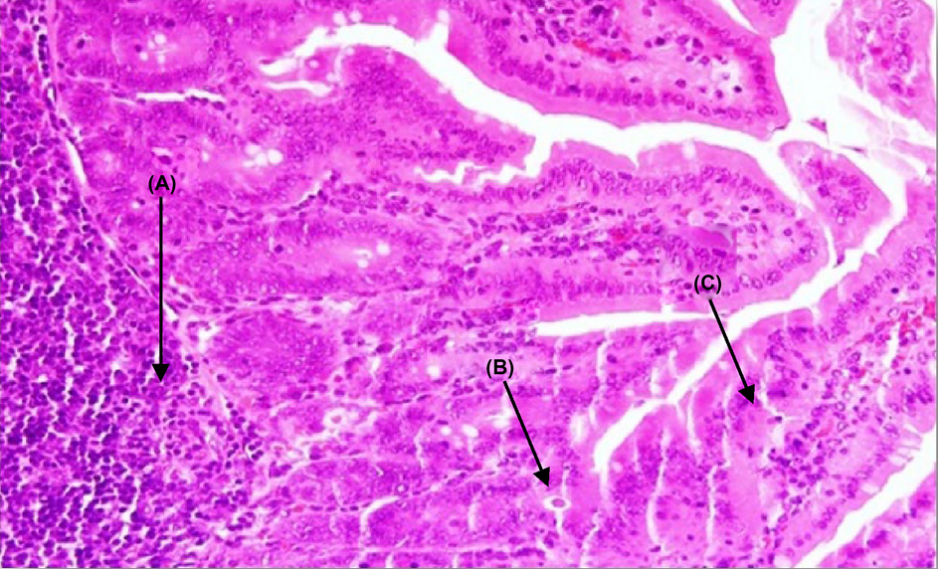
A section of jejunum from a mouse In experimental group IA on the tenth day, showing (**A**) inflation in Bayer cells, (**B**) increase in cellular infiltration, and (**C**) presence of some trophozoites and disappearance of trophozoites (H & E, 200×).

**Figure 6:**
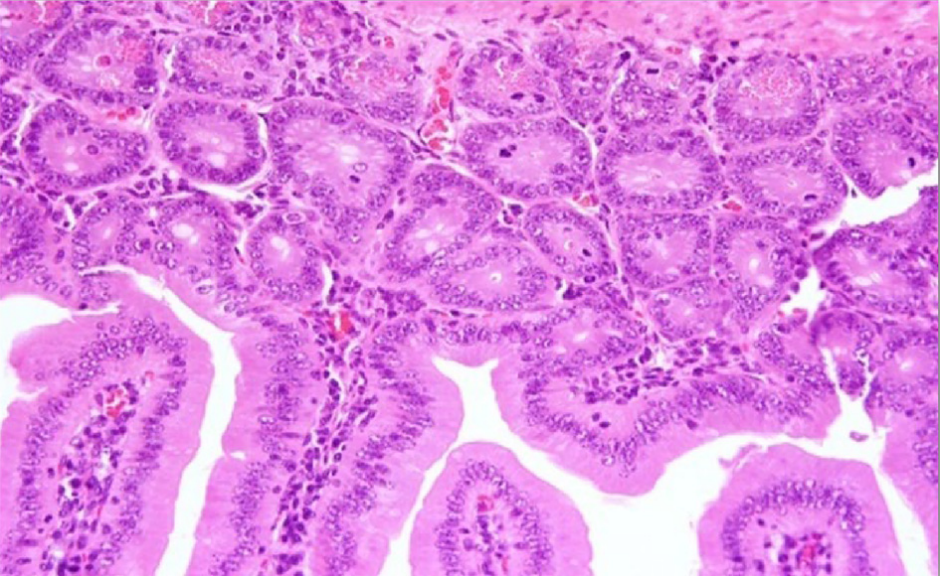
A section of jejunum from a mouse In experimental group IA on the 25th day, showing the return of the villi to its natural shape and composition (H & E, 200×).

The examination of the jejunum segments showed satisfactory changes on the 10th day of infection (Figure 5). There was a high increase in cellular infiltration in the central pulp, and oedema increased with a hyperplasic Peyer’s patch and disappearance of trophozoites. On the 25th day (Figure 6), the villi recovered their normal structure without any noticeable changes.

## Discussion

Giardiasis is one of the most common causes of diarrhea and malnutrition worldwide and has varying symptoms [26]. Pharmaceutical treatment remains controversial among doctors, researchers, and international health authorities because of its serious side effects and its failure to treat many patients. This is why we need to seek effective and safe alternative treatment [27]. Some studies show that the treatment of giardiasis using alternative therapy can reduce the symptoms of the disease and helps to get rid of the parasite [28].

We chose this topic to study in order to shed more light on the effectiveness of biotherapy, specifically with regards to beneficial bacteria eliminating the parasite. The study was performed on small albino mice as a pilot model of infection very similar to *G. lamblia* infection in humans. Young mice were selected as they are more susceptible to infection and virtually free from parasitic infections [29]. Results showed an infection rate of 100% in mice from the seventh day after exposure to the parasite. This is consistent with their known high susceptibility to *G. lamblia* infection [29].

In the current study, we used strains of probiotics have been previously used in studies to some effect, supporting their use in treatment. These bacteria help to eliminate the intestinal parasites that may exist with them in the same environmental space, possibly by improving the functions of the immune system and via other mechanisms [30–32]. Additionally, these bacteria have had an impact in eliminating some other parasites such as *Cryptosporidium* sp. [33].

Our study showed that there was a decrease in the number of cysts in the stool samples of experimental group IB with significant differences among days 10, 15, and 20. ELISA indicated a gradual decrease in parasite antigen in the stool, with very high significant differences between day 7 and the remaining test days.

The results of *G. lamblia* antigen detection in the stool by ELISA showed that cure rate using probiotics reached 87.5%, while no cure was achieved in the control groups. This cure rate is similar to that of metronidazole treatment. This is significant, as this therapy appears to be a viable option in cases where metronidazole is banned. In study of Magda et al., 2020 [34], stated that using of Acidophilus probiotic mixture of *L. acidophilus*, L. *bulgaricus, L. sylvarius, L. brevis*, and *B. bifidium* versus metronidazole against giardiasis in infected mice, showed less patent period decrease in infection intensity and significant increase in reduction cyst shedding at 10, 13, and 17th post-infection days as compared with control (*P*0.01), but no significant difference between probiotics treated group compared with metronidazole (*P*0.05).

The rate of prevention reached 30% in experimental group IA, while there was no protection detected in the rest of the groups (100% of subjects were infected with *G. lamblia*).

Our results demonstrate the effectiveness of using beneficial bacteria as a treatment against the parasite *G. lamblia*. These results are consistent with previous research that beneficial bacteria have a protective role against intestinal giardiasis, improving resistance to and preventing infection by this parasite. This may be due to many factors, including interactions between beneficial bacteria and the parasite [30,32]. Some have suggested that the eradication of *G. lamblia* parasites is due to the effect of *Lactobacilli* bacteria on the immune system, which helps to get rid of the parasite [35]. It is considered that the use of beneficial bacteria can be expanded to include alternative strategies in the prevention of extensive spread of intestinal parasitic infections [36].

In the untreated control group, the intestinal segments showed a high density of trophozoites in the intestinal cavity, between the villi, and adherent to the surface of the epithelial cells. There were some changes in the shape of the villi including shortening and flattening of the top, as well as increased cellular infiltration and the appearance of hyperplasia. These results are consistent with studies reporting similar changes in the small intestine during *G. lamblia* infection [37]. These changes can often be interpreted as a non-qualitative response to gastrointestinal mucosa irritation due to adhesion of trophozoites or the release of cytotoxicity. Additionally, the large numbers of bacteria and fungi that accompany *G. lamblia* infection may play a role, along with cytokines involved in the inflammation of intestinal mucosa [38].

In the present study, there was a marked improvement in the histological changes in experimental group IB; the presence of trophozoites was significantly lower on the tenth day compared with the control group and the pathological changes were fewer. Almost all of these changes disappeared on the 25th day except for a slight increase in cellular infiltration. In experimental group IA, the tissues recovered their normal structure without any noticeable changes.

The present study indicates the effectiveness of beneficial bacteria in the treatment of giardiasis and in the improvement of histologic changes associated with this disease, which is likely to improve the symptoms and health of the patient. Additionally, the greater improvement in group IA compared with IB confirms the role of beneficial bacteria in the prevention of the disease.

The most commonly consumed probiotics belong to the genus *Lactobacillus* and *Bifidobacterium*. Mechanisms by which probiotics might improve host health include immune function augmentation through reinforcing mucosal barrier function, reducing mucosal transfer of luminal organisms and metabolites to the host, increasing mucosal antibody production, strengthening epithelia integrity, and direct antagonism of pathogenic microorganisms [39]. Some bacteria genera, such as *Lactobacillus* and *Bifidobacterium*, have a direct relation with immune response stimulation because they enhance IgA production and secretion through an alteration of the cytokine milieu in the gut mucosa, induce epithelial cell expression of TGFβ and IL-10 as well as IL-6, which potentiate IgA production, and induce/augment the expression of polymeric Ig receptors on the basolateral surface of intestinal epithelial cells [40].

These results are consistent with studies indicating that there is a marked reduction in harmful pathological changes after treatment with beneficial bacteria. In addition, it appears that such bacteria help modify immunological properties and have a significant role in the adjustment of the mucous membrane of the intestines. Treatment with beneficial bacteria should be thought of as a primary treatment and not only as a promoter of other parasites, but also as a preventive measure, especially in cases of traveler’s diarrhea [41,42].

Our results are consistent with those of another study conducted on the effectiveness of the plant *Piper betle* against *G. lamblia*, where there was a significant reduction in the number of cysts indicating that water extract of the plant can be used as a therapy against this disease [43].

## Conclusions

The use of probiotics as a natural and safe product for enhancing intestinal health has been confirmed through the present study. Probiotics containing bacterial mixtures traditionally contain viable microorganisms of *L. acidophilus, B. bifidum*, and *L. helveticus*, and have a beneficial and significant effect in the prevention and treatment of giardiasis in mice. Further studies are still required before entry into clinical trials as the mechanism of action on molecular bases.

## Data Availability

The datasets used and/or analyzed during the current study are available from the corresponding author on reasonable request.

## Abbreviations

CFS: chronic fatigue syndrome
ELISA: enzyme-linked immunosorbent assay
GNC: general nutrition centers
IBS: irritable bowel syndrome
Th17: T-helper 17
Treg: T-regularoty
