# Correction: Preventive role of probiotic bacteria against gastrointestinal diseases in mice caused by *Giardia lamblia*

**DOI:** 10.1042/BSR-2020-4114_COR

**Published:** 2021-06-02

**Keywords:** ELISA, histopathological studies, probiotics

This article is being corrected following notification from one of our readers alerting Bioscience Reports to duplicated sections within Figures 2-5. The Editorial Office contacted the authors and were provided with original images and raw data which did not contain the areas of concern raised by the reader. Following investigation by the Editorial Office, it was identified that the version of files accepted for publication on 12 February 2021 did not contain the image duplications that were identified post-publication. It has since been discovered that the duplications were introduced by an external production vendor during the production process prior to publication in order to achieve a coherent image style (i.e. modification of panel labelling within a figure), and thus first appeared in the Version of Record published by Bioscience Reports on 26 February 2021.

**Figure 2:**
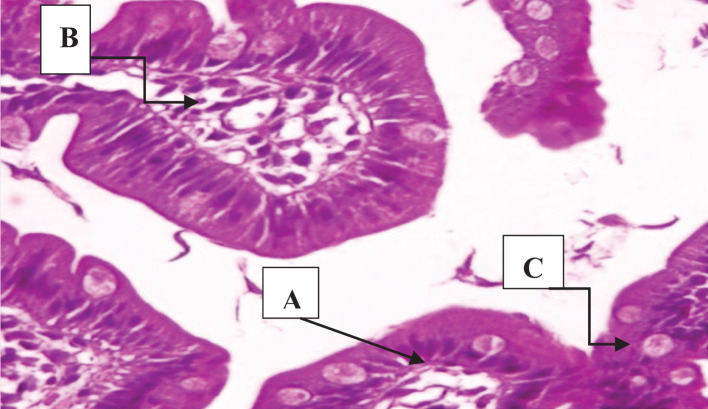
A section of jejunum from a mouse In control group IIA, (**A**) integration in some villi, (**B**) cellular infiltration, and (**C**) showing the density of the trophozoites between the cells and their adhesion to the mucous surface of cells (H & E, 400×).

**Figure 3:**
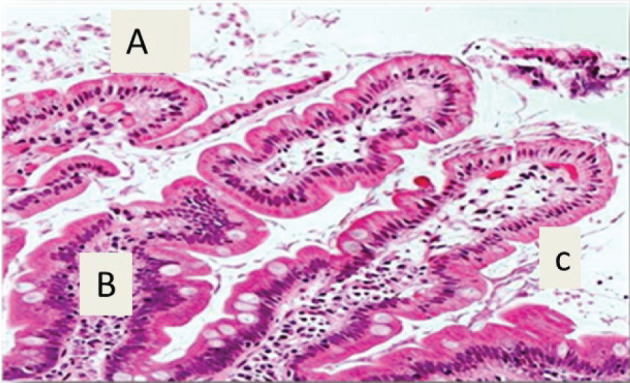
A section of jejunum from a mouse In experimental group IB, showing (**A**) integration in some villi, (**B**) increase in cellular infiltration, and (**C**) presence of some trophozoites (H & E, 400×).

**Figure 4:**
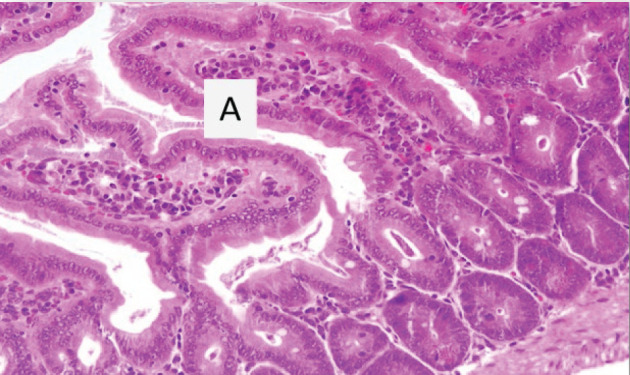
A section of jejunum from a mouse In experimental group IB on the 25th day, showing the (**A**) complete disappearance of trophozoites and that the villi is restored to its normal shape (H & E, 20×).

**Figure 5:**
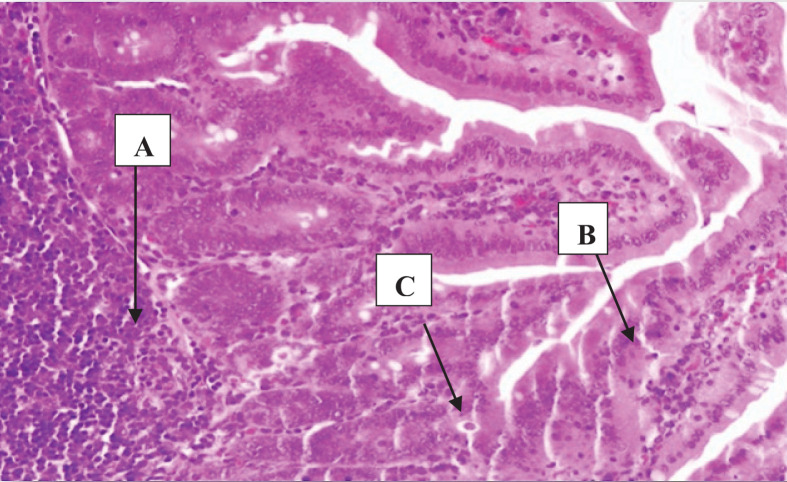
A section of jejunum from a mouse In experimental group IA on the tenth day, showing (**A**) inflation in Bayer cells, (**B**) increase in cellular infiltration, and (**C**) presence of some trophozoites and disappearance of trophozoites (H & E, 200×).

The correct version of Figures 2-5 as originally submitted by the authors and present in the accepted manuscript are included in this Correction. The Editorial Office apologises to both the authors of this paper and readers that such duplications were introduced. Immediate workflow changes have taken place within our production processes to prevent such errors occurring in the future.

